# Utilization of T1-Mapping for the pelvic and thigh muscles in Duchenne Muscular Dystrophy: a quantitative biomarker for disease involvement and correlation with clinical assessments

**DOI:** 10.1186/s12891-022-05640-y

**Published:** 2022-07-16

**Authors:** Fei Peng, Huayan Xu, Yu Song, Ke Xu, Shuhao Li, Xiaotang Cai, Yingkun Guo, Lianggeng Gong

**Affiliations:** 1 Department of Medical Imaging center, The Second Affiliated Hospital of Nanchang University, Minde Road No. 1, Nanchang, 330006 Jiangxi Province China; 2 Department of Radiology, Key Laboratory of Obstetric and Gynecologic and Pediatric Diseases and Birth Defects of Ministry of Education, West China Second University Hospital, Sichuan University, 20# Section 3 South Renmin Road, Chengdu, 610041 Sichuan Province China; 3 Department of Pediatrics Neurology, West China Second University Hospital, Sichuan University, 20# Section 3 South Renmin Road, Chengdu, 610041 Sichuan Province China

**Keywords:** Magnetic resonance imaging, T1-mapping, T1, Duchenne Muscular Dystrophy, Skeletal muscle

## Abstract

**Background:**

Little is known about the disease distribution and severity detected by T1-mapping in Duchenne muscular dystrophy (DMD). Furthermore, the correlation between skeletal muscle T1-values and clinical assessments is less studied. Hence, the purposes of our study are to investigate quantitative T1-mapping in detecting the degree of disease involvement by detailed analyzing the hip and thigh muscle, future exploring the predicting value of T1-mapping for the clinical status of DMD.

**Methods:**

Ninety-two DMD patients were included. Grading fat infiltration and measuring the T1-values of 19 pelvic and thigh muscles (right side) in axial T1-weighted images (T1WI) and T1-maps, respectively, the disease distribution and severity were evaluated and compared. Clinical assessments included age, height, weight, BMI, wheelchair use, timed functional tests, NorthStar ambulatory assessment (NSAA) score, serum creatine kinase (CK) level. Correlation analysis were performed between the muscle T1-value and clinical assessments. Multiple linear regression analysis was conducted for the independent association of T1-value and motor function.

**Results:**

The gluteus maximus had the lowest T1-value, and the gracilis had the highest T1-value. T1-value decreased as the grade of fat infiltration increased scored by T1WI (*P* < 0.001). The decreasing of T1-values was correlated with the increase of age, height, weight, wheelchair use, and timed functional tests (*P* < 0.05). T1-value correlated with NSAA (r = 0.232-0.721, *P* < 0.05) and CK (r = 0.208-0.491, *P* < 0.05) positively. T1-value of gluteus maximus, tensor fascia, vastus lateralis, vastus intermedius, vastus medialis, and adductor magnus was independently associated with the clinical motor function tests (*P* < 0.05). Interclass correlation coefficient (ICC) analysis and Bland-Altman plots showed excellent inter-rater reliability of T1-value region of interest (ROI) measurements.

**Conclusion:**

T1-mapping can be used as a quantitative biomarker for disease involvement, further assessing the disease severity and predicting motor function in DMD.

**Supplementary Information:**

The online version contains supplementary material available at 10.1186/s12891-022-05640-y.

## Introduction

Duchenne muscular dystrophy (DMD) is one of the most common neuromuscular disorders (NMDs), affecting approximately 1 of 3500-5000 male births worldwide [1, 2]. DMD is characterized by progressive, irreversible intramuscular fat infiltration that induces muscle weakness [3]. The motor function gradually develops from the initial gait abnormalities to loss of ambulation and ultimately relies on a wheelchair [4].

Although the disease remains incurable so far, a comprehensive and accurate assessment of disease status is crucial for implementing of treatment measures that can delay the disease progression and improve the life quality of DMD patients [5, 6]. Clinical assessment methods such as manual muscle testing, range of joint motion, timed functional tests, motor function scales have been widely applied to evaluate the disease status of DMD patients [7–9]. However, the main disadvantages of these methods are unquantifiable, low repeatability, and insufficient accuracy. Also the individual muscles are not be evaluated [1, 7]. In addition, the serum creatine kinase (CK) level may not be linearly related to the disease progression, especially at the end stage of the disease. Thereby it is considered unreliable [10]; muscle biopsies may have a bias due to the small sampling site, and repeated muscle biopsy is not practical especially for children [11]. Hence, exploring more repeatable and accurate means is necessary to quantify the disease status.

Magnetic resonance imaging (MRI) can evaluate the anatomical shape, fat infiltration, edema, fibrosis, fiber orientation, and metabolism of skeletal muscles through various technical sequences, which suggests that it is a robust biomarker candidate for monitoring the pathological process and disease status in NMDs [12]. Multiple consensus guidelines have proposed quantitative MRI as an imaging biomarker for DMD [12–14]. As the most crucial feature in DMD, the degree of muscle fat infiltration correlates with muscle function, and changes in fat content precede changes in clinical process [12, 15]. Therefore, a comprehensive assessment of fat infiltration is essential to help understand the disease status. As a non-invasive quantitative MRI technique, T1-mapping has been increasingly applied to monitor chronic fatty degenerations of lower limb muscles within the course of NMDs; e.g. Becker muscular dystrophy (BMD) or UDP-N-acetylglucosamine 2-epimerase/N-acetylmannosamine kinase (GNE) myopathy [16–18]. Notably, T1-mapping presents an optimistic clinical application prospect in monitoring fat infiltration of skeletal muscles, which may provide a biomarker for further understanding of disease status [16, 18]. Nevertheless, little is known about the disease distribution and severity detected by T1-mapping in DMD. Furthermore, the correlation between T1-values of skeletal muscles and clinical assessments is also less studied.

Therefore, the purposes of our study are to investigate quantitative T1-mapping in detecting the degree of disease involvement by detailed analyzing the hip and thigh muscle, future exploring the predicting value of T1-mapping for the clinical status of DMD.

## Materials and methods

### Patient recruitment

We had already established a prospective cohort of DMD, and DMD patients confirmed through genetic analysis and/or muscle biopsy in this clinical cohort will routinely undergo MRI scans of the hip and thigh. This prospective study was approved by the institutional review board, and written informed consent was required from all participants and/or guardians before study participation.

### Imaging acquisition

Imaging evaluations comprising semi-quantitative T1-weighted images (T1WI) and quantitative T1-mapping were performed from the iliac crest to the middle thigh using a 3.0 T imaging system (Siemens Magnetom Skyra, a Tim and Dot System, Healthineers) with a Body18 channel coil. Since the T1-value of skeletal muscle can be influenced by exercise or activity [17], restrictions on excessive motion or exercise such as running, hiking and long-distance walking, etc., were required before the MRI scan. No intravenous contrast agent was used.

Axial turbo spin-echo T1WI without fat suppression were obtained with the following parameters: Voxel size = 0.6 × 0.6 × 6 mm^3^, TR = 813 ms, TE = 12 ms, FOV = 333 × 281 mm, Slice thickness = 6 mm, Slice gap = 2.4 mm, Tacq = 65 s.

Axial T1-maps were obtained using the shortened modified look-locker inversion (MOLLI) recovery sequence. After reversing the recovery pulse, the acquisition of single excitation is repeated every 2 seconds. Eight points every slice were used to fit the T1 curve. The protocol was modified by acquiring 5 images after the first inversion, followed by a 180 ms pause and then acquire 3 images after the second inversion. The parameters were as follows: Voxel size = 1.6 × 1.6 × 6 mm^3^, TR = 279.12 ms, TE = 1.1 ms, FOV = 341 × 401 mm, flip angle (FA) = 35°, Slice thickness = 6 mm, Slice gap = 1.8 mm, Tacp = 54 s.

### Image Analysis

Two pediatric musculoskeletal radiologists performed all data evaluation and measurement independently (with 5 and 13 years of experience, respectively) using Siemens MR-Post-Processing workstation (Syngo. Via). The patient information in the images was hidden to minimize learning bias.

**Fig. 1:**
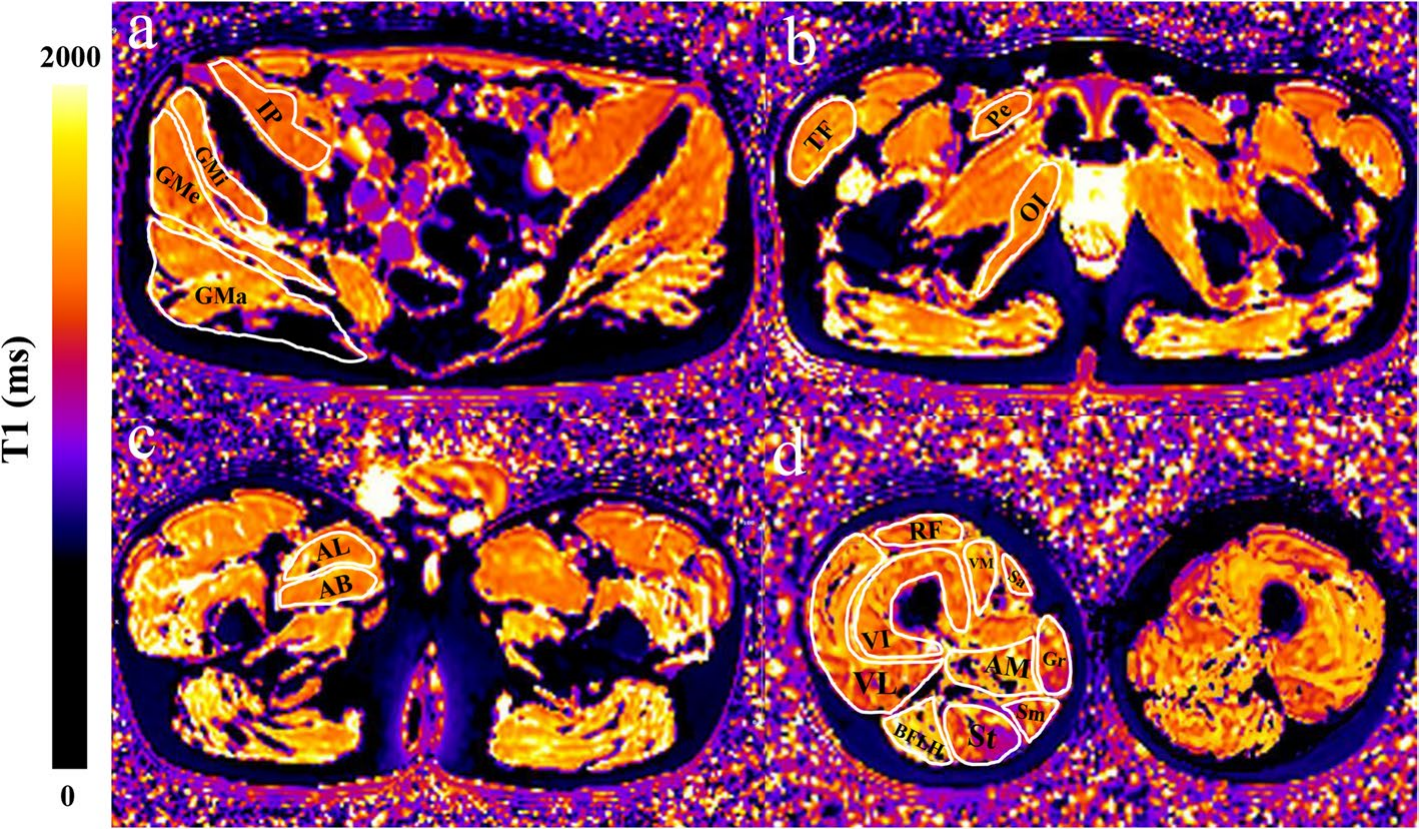
Images in a 6-year-old DMD boy. The axial T1-maps with colors corresponding to a T1-value range were on the left column. Four cross-section levels [(1) level near the sciatic foramen (**a**); (2) level near the greater trochanter-ischial tuberosity (**b**); (3) level near the closer proximal part of the femoral diaphysis (**c**); (4) approximately 5 cm below level (3) (**d**)] were chosen. Evaluation of individual muscle at each level as follows: Level (1) (**a**), Gluteus maximus (GMa), Gluteus medius (GMe), Gluteus minimus (GMi), Iliopsoas (IP); Level (2) (**b**), Tensor fascia (TF), Obturator internus (OI), Pectineus (Pe); Level (3) (**c**), Adductor longus (AL), Adductor brevis (AB); Level (4) (**d**), Rectus femoris (RF), Vastus lateralis (VL), Vastus intermedius (VI), Vastus medialis (VM), Gracilis (Gr), Sartorius (Sa), Adductor magnus (AM), Semitendinosus (St), Semimembranosus (Sm), Biceps femoris long head (BFLH). A total of 19 muscles on the right side were included for assessment

Four representative cross-section levels [(1) level near the sciatic foramen; (2) level near the greater trochanter-ischial tuberosity; (3) level near the closer proximal part of the femoral diaphysis; (4) approximately 5 cm below level (3)] that contained larger area of visible muscle with the excellent distinction of different muscle compartments were chosen [19, 20] (Fig. 1). Nineteen muscles (gluteus maximus, gluteus medius, gluteus minimus, iliopsoas, tensor fascia, obturator internus, pectineus, rectus femoris, vastus lateralis, vastus intermedius, vastus medialis, gracilis, sartorius, adductor longus, adductor brevis, adductor magnus, semitendinosus, semimembranosus, biceps femoris long head) in the right side of the pelvic girdle and right thigh were assessed (Fig. 1).

Visual grading of axial T1WI through the 5-point modified Mercuri scale of fat infiltration (0 = normal, 1 = mild fatty streaks, 2 = mild fat infiltration < 30%, 3 = moderate fat infiltration between 30 and 60%, 4 = severe fat infiltration > 60%) [20, 21]. If two observers disagreed in grading, a third observer (with over 20 years of experience in pediatric radiology) provided an independent grading to break the deadlock.

The T1-maps were color coded pixel by pixel with colors corresponding to a range of T1-values. The region of interest (ROI) was obtained by manually tracing the outline of the individual muscle. The ROI size was determined by using the individual muscle size on the axial images. Placing the ROI of each muscle on the T1-maps automatically generated a mean T1-value for each muscle. Finally, the average value measured by the two radiologists was taken as the T1-value of each muscle.

### Clinical Assessments

All included patients received clinical assessments by a pediatric neurologist with particular expertise in neuromuscular diseases blinded to the MRI findings. Clinical assessments, including the participant’ age, height, weight, body mass index (BMI), wheelchair use, NorthStar ambulatory assessment (NSAA) score, timed functional tests (10-m run/walk, Gowers manoeuvre, 4-stair climb, 4-stair descend), and serum CK level was performed within 3 days of MRI. The Gowers manoeuvre was the time required for the patient to rise from a sitting position on the floor to standing [20]. The NSAA used a 17-item rating scale, and each item was 0-2 points, with a total score being 34 points [22]. The higher the total score of NSAA, the better the child’s motor function.

### Statistical Analysis

The Shapiro-wilk test was used to evaluate the normality of the data distribution. The individual muscle T1-value among different grades of fat infiltration was compared by test for trend. The Spearman method with Benjamini-Hochberg correction was used to assess the correlation between T1-values, the Mercuri scale and clinical assessments. Mann-Whitney test for individual muscle T1-value and Mercuri scale was performed in DMD patients who used wheelchairs or not. Multiple linear regression analysis was used to determine the independent association of T1-value and clinical motor function. The above results were considered statistically significant when the *P* < 0.05. Bland-Altman plots and interclass correlation coefficient (ICC) were used to determine the inter-rater reliability of the T1-value ROI measurement. All statistical analyses were conducted with SPSS version 22.0 and MedCalc Version 20.011.

## Results

### Participant Characteristics

**Fig. 2:**
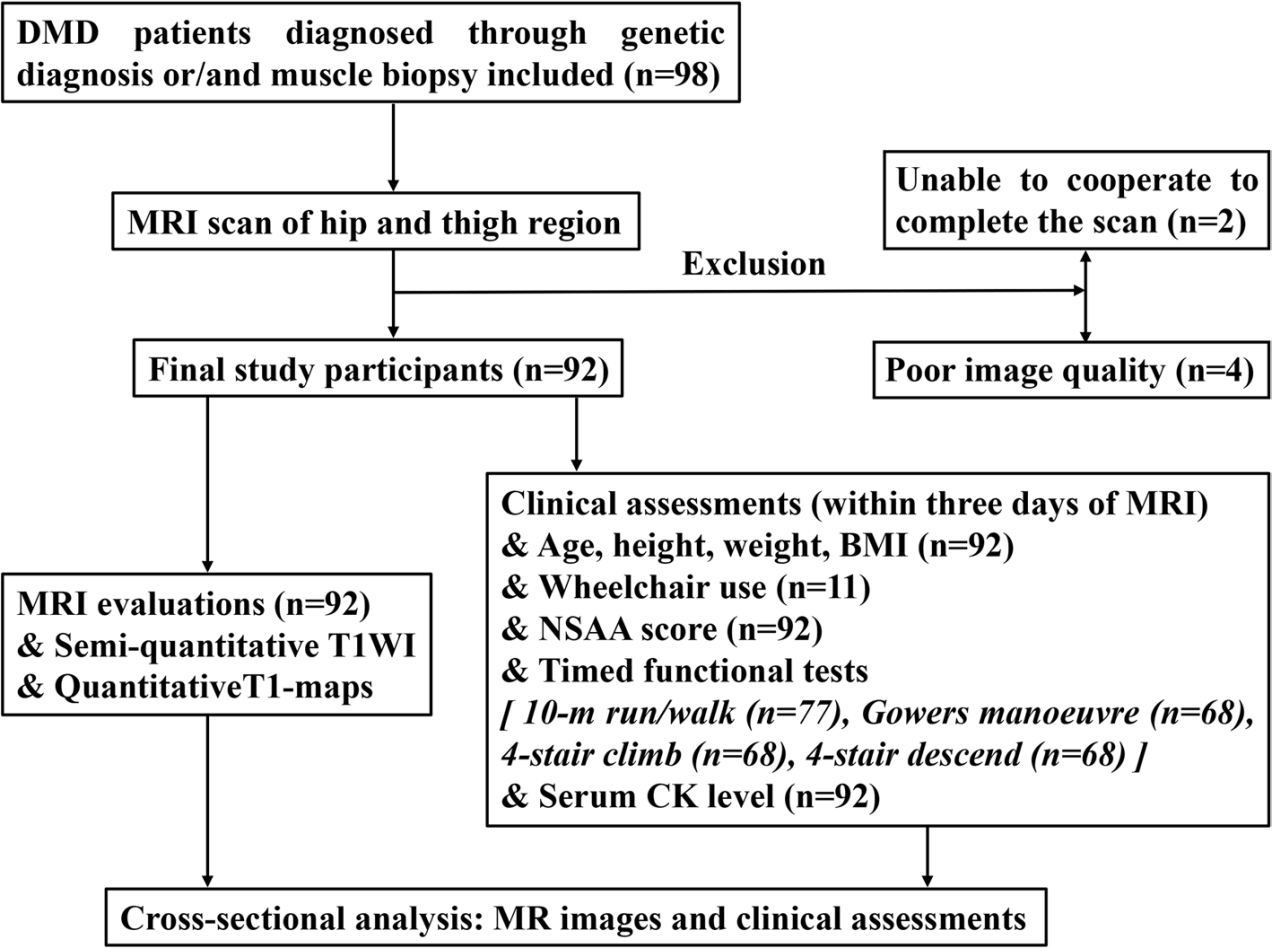
Flowchart showed selection and assessment of study participants. DMD, duchenne muscular dystrophy; T1WI, T1-weighted images; NSAA, northstar ambulatory assessment; CK, creatine kinase

Between May 2020 and July 2021, written informed consent was obtained from 98 subjects and MRI scans were performed. Two participants could not cooperate to complete the scan, and four with poor image quality were excluded. Finally, 92 subjects (mean age was 8.78 ± 2.06 years, range 5-15 years) were included in this study (Fig. 2). The characteristics of the included 92 participants are shown in Table 1. Approximately 12.0% of patients (11 of 92) used a wheelchair in the study group. Since the timed functional tests can only be carried out in cooperative patients with mild to moderate dysfunction, the 10-m run/walk, Gowers manoeuvre, 4-stair climb, 4-stair descend were obtained in 77, 68, 68, 68 patients, respectively. NSAA scores and serum CK levels were acquired in all 92 patients.

**Table 1:** Characteristics of the study group

Parameters	Number (Total 92)	Mean ± SD (Range)
Age (years)	92	8.78 ± 2.06 (5-15)
Height (cm)	92	125.00 ± 12.55 (90-168)
Weight (kg)	92	28.38 ± 9.96 (12-75)
BMI (kg/m^2^)	92	17.79 ± 3.77 (7.80-30.18)
Wheelchair use / not used	11 / 81	
NSAA score	92	16.79 ± 10.98 (0-34)
10-m run/walk (s)	77	7.40 ± 5.37 (3.1-27.21)
Gowers manoeuvre (s)	68	6.16 ± 6.45 (1.12-36.28)
4-stair climb (s)	68	4.32 ± 4.08 (0.81-21.03)
4-stair descend (s)	68	3.49 ± 4.52 (0.91-30.03)
CK (reference value 39-192 U/L)	92	10,982.73 ± 6303.23 (2190-34,715)

*SD* Standard deviation, *BMI* Body mass index, *NSAA* Northstar ambulatory assessment, *CK* Creatine kinase

### Disease severity and distribution on T1WI and T1-maps

**Fig. 3:**
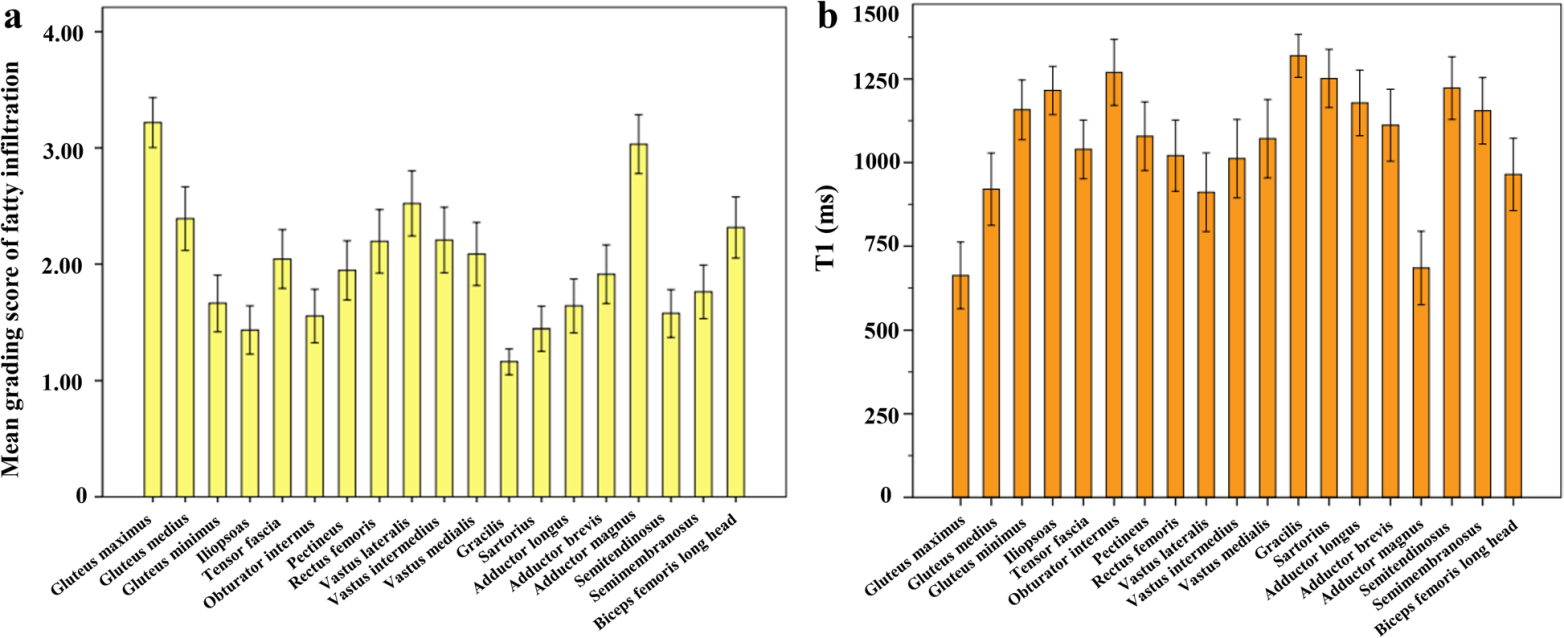
The bar chart showed the mean score of all grades of fat infiltration for each pelvic and thigh muscle on T1-weighted images (**a**). The gluteus maximus achieved the highest mean score of fat infiltration, followed by the adductor magnus, the gracilis muscle presented the lowest mean score. Mean T1-value distribution of 19 pelvic and thigh muscles was exhibited in **b**. The gluteus maximus has the lowest T1-value, followe by the adductor magnus, the gracilis muscle has the highest T1-value

The bar chart showed the mean score of fat infiltration (Fig. 3a) and mean T1-value (Fig. 3b) for individual muscle. The gluteus maximus achieved the highest mean score of fat infiltration and had the lowest T1-value, followed by the adductor magnus, the gracilis muscle showed the lowest mean score of fat infiltration and had the highest T1-value.

**Fig. 4:**
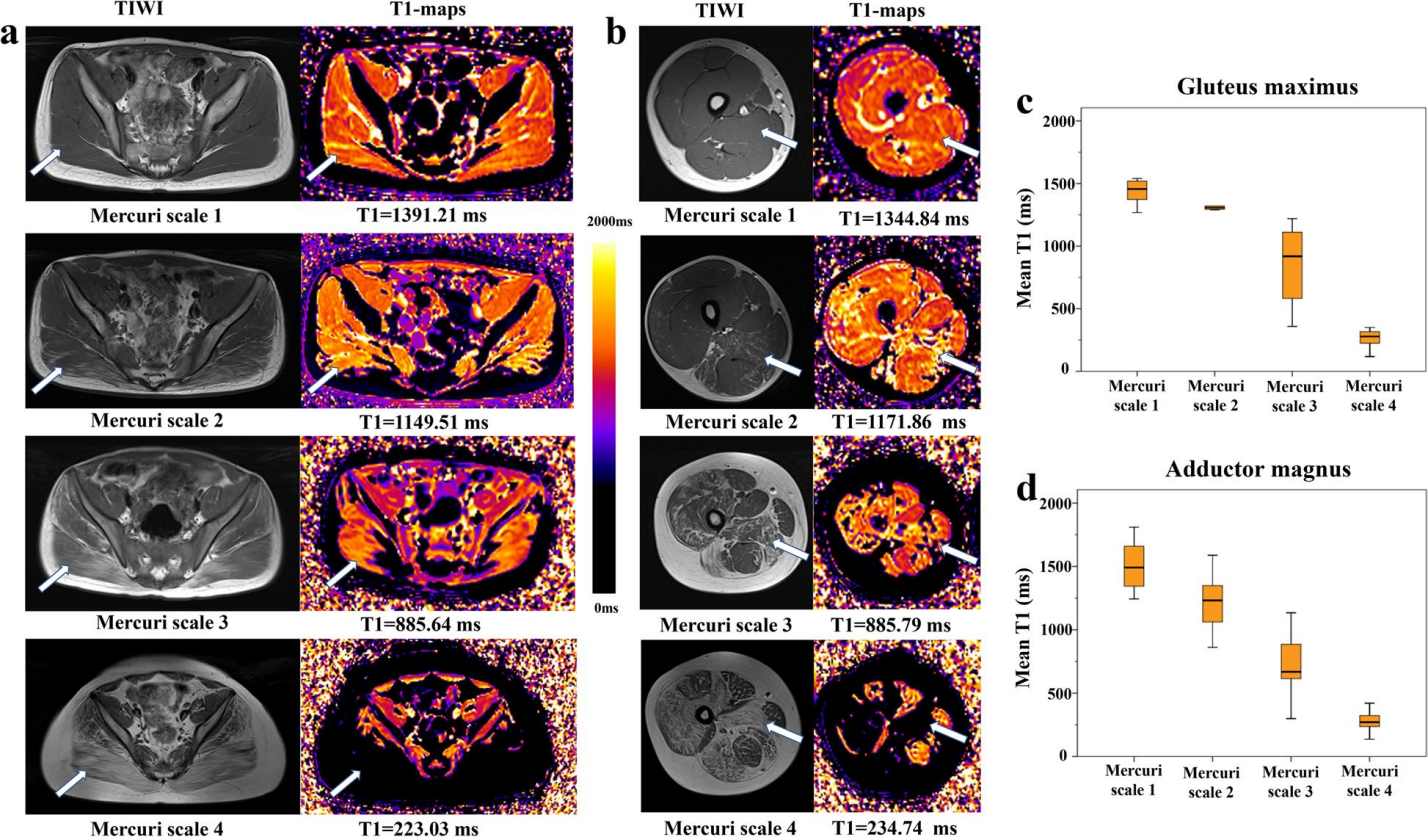
The T1-maps corresponding to a different grades of fat infiltration on T1-weighted images (T1WI) of gluteus maximus (white arrow in the left column) in different DMD patients: Mercuri scale 1 → T1 = 1391.21 ms, Mercuri scale 2 → T1 = 1149.51 ms, Mercuri scale 3 → T1 = 885.64 ms, Mercuri scale 4 → T1 = 223.03 ms (**a**). The T1-maps corresponding to different grade of fat infiltration on T1WI of adductor magnus (white arrow in right column): Mercuri scale 1 → T1 = 1344.84 ms, Mercuri scale 2 → T1 = 1171.86 ms, Mercuri scale 3 → T1 = 885.79 ms, Mercuri scale 4 → T1 = 234.74 ms (**b**). The box plots showed that the T1-value of gluteus maximus (**c**) and T1-value of adductor magnus (**d**) decreased with the increase of fat infiltration grade

Table 2 listed the T1-values of individual muscle and the number of patients corresponding to the different fat infiltration grade groups. There appears no Mercuri scale 0 muscles, which suggesting widespread muscle involvement even at relatively early disease stages. As the grade of fat infiltration increased, the T1-value of individuals all decreased (all *P-*trend < 0.001). The T1-maps corresponding to a different grade of fat infiltration of gluteus maximus and adductor magnus muscle, and the box plots of gluteus maximus and adductor magnus muscle T1-value decreased with the increased of fat infiltration grade were taken as examples (Fig. 4).

**Table 2:** The T1-values (M ± SD) of individual muscle and the number of patients among different Mercuri scale of fat infiltration

T1-mapping	Muscle	Mercuri scale (total *n* = 92)				
		1 (n)	2 (n)	3 (n)	4 (n)	*P* for trend
T1-value (ms)	Gluteus maximus	1438.0 ± 98.9 (8)	1280.7 ± 101.3 (18)	833.6 ± 274.5 (13)	293.9 ± 114.14 (53)	<0.001
	Gluteus medius	1429.4 ± 146.2 (38)	1086.4 ± 257.5 (11)	747.2 ± 298.3 (12)	304.2 ± 58.5 (31)	<0.001
	Gluteus minimus	1377.3 ± 146.5 (66)	1138.6 ± 142.8 (7)	627.5 ± 441.2 (2)	349.7 ± 102.9 (17)	<0.001
	Iliopsoas	1354.0 ± 141.4 (76)	1059.2 ± 252.2 (2)	752.4 ± 102.6 (4)	380.8 ± 141.2 (10)	<0.001
	Tensor fascia	1358.4 ± 130.3 (46)	1122.2 ± 164.1 (16)	738.0 ± 229.2 (10)	390.4 ± 181.8 (20)	<0.001
	Obturator internus	1494.7 ± 207.4 (71)	1070.5 ± 71.4 (4)	589.4 ± 247.9 (3)	330.3 ± 93.1 (14)	<0.001
	Pectineus	1409.3 ± 245.0 (52)	1151.8 ± 183.0 (12)	676.9 ± 278.2 (9)	317.6 ± 74.9 (19)	<0.001
	Rectus femoris	1423.7 ± 181.7 (46)	1243.9 ± 100.8 (8)	835.2 ± 244.9 (12)	325.8 ± 202.3 (26)	<0.001
	Vastus lateralis	1494.7 ± 193.5 (35)	1219.5 ± 234.1 (11)	734.2 ± 218.0 (9)	310.5 ± 126.4 (37)	<0.001
	Vastus intermedius	1495.0 ± 172.7 (47)	1006.9 ± 271.3 (9)	771.8 ± 266.8 (6)	304.2 ± 132.0 (30)	<0.001
	Vastus medialis	1504.0 ± 194.2 (50)	1125.5 ± 191.1 (9)	757.7 ± 422.8 (8)	287.1 ± 105.1 (25)	<0.001
	Gracilis	1416.2 ± 177.5 (79)	1038.6 ± 157.6 (5)	633.8 ± 138.4 (5)	375.5 ± 48.5 (3)	<0.001
	Sartorius	1418.9 ± 182.2 (72)	1239.1 ± 123.8 (7)	649.5 ± 274.5 (3)	234.9 ± 61.3 (10)	<0.001
	Adductor longus	1436.2 ± 180.2 (65)	998.7 ± 303.0 (9)	584.6 ± 110.3 (3)	288.2 ± 94.9 (15)	<0.001
	Adductor brevis	1460.0 ± 180.2 (54)	1223.0 ± 236.6 (10)	574.8 ± 336.4 (10)	303.2 ± 60.5 (18)	<0.001
	Adductor magnus	1493.4 ± 182.1 (18)	1191.1 ± 208.9 (13)	699.5 ± 255.2 (9)	275.6 ± 70.3 (52)	<0.001
	Semitendinosus	1454.1 ± 196.8 (64)	1156.1 ± 189.9 (11)	647.2 ± 150.1 (5)	289.3 ± 97.0 (12)	<0.001
	Semimembranosus	1445.3 ± 206.2 (57)	1140.6 ± 246.6 (13)	519.2 ± 234.9 (9)	335.5 ± 126.3 (13)	<0.001
	Biceps femoris long head	1473.3 ± 180.4 (37)	1098.5 ± 186.1 (16)	695.5 ± 196.0 (12)	307.3 ± 88.3 (27)	<0.001

The test for trend was performed with a polynominal contrast procedure, *P* < 0.05, statistical significance. M ± SD, mean ± standard deviation

### Correlation between T1-value, Mercuri scale of fat infiltration and clinical assessments

Table 3 presented the correlation between individual muscle T1-value and clinical assessments. Negative correlations were found between T1-value and the participants’ age (r = − 0.297 to − 0.629, all *P* <0.05), height (r = − 0.244 to − 0.571, all *P* <0.05), weight (*P* value of 17 muscles were <0.05, r = − 0.283 to − 0.495), BMI (*P* value of 6 muscles were <0.05, r = − 0.255 to − 0.286), 10-m run/walk (*P* value of 12 muscles were <0.05, r = − 0.268 to − 0.605), Gowers manoeuvre (*P* value of 9 muscles were <0.05, r = − 0.366 to − 0.705), 4-stair climb (*P* value of 9 muscles were <0.05, r = − 0.308 to − 0.668), 4-stair descend (*P* value of 8 muscles were <0.05, r = − 0.333 to − 0.527). Positive correlations were discovered between T1-value and NSAA (r = 0.232 to 0.721, all *P* <0.05), serum CK level (*P* value of 14 muscles were <0.05, r = 0.292 to 0.491). Notably, the T1-value of gluteus maximus, gluteus medius, tensor fascia, pectineus, rectus femoris, vastus lateralis, vastus intermedius, vastus medialis, adductor magnus, biceps femoris long head showed a correlation (*P*<0.05) with both NSAA and timed functional tests. Besides, most of the correlations between T1-values and clinical assessments were moderate or high [r (absolute value) = 0.404 to 0.721, *P* < 0.01]. Although some of the correlations appeared to be relatively weak (r = − 0.394 to 0.396), they were still statistically significant (*P* < 0.05). Especially in the correlations between T1-values and NSAA, except the gracilis and sartorius showed a relatively weak correlation (r = 0.232 to 0.330, *P* < 0.05), the T1-values of the remaining 17 muscles all showed moderate to strong correlation with NSAA (r = 0.419 to 0.721, *P* < 0.01).

**Table 3:** Spearman correlation between individual muscle T1-value and clinical assessments

T1-value of individual muscle																			
	GMa	GMe	GMi	IP	TF	OI	Pe	RF	VL	VI	VM	Gr	Sa	AL	AB	AM	St	Sm	BFLH
Age (n = 92)	−.334^**^	−.623^**^	−.445^**^	−.347^**^	−.515^**^	−.461^**^	−.587^**^	−.507^**^	−.577^**^	−.629^**^	−.566^**^	−.297^**^	−.379^**^	−.457^**^	−.482^**^	−.418^**^	−.505^**^	−.535^**^	−.616^**^
Height (n = 92)	−.404^**^	−.529^**^	−.354^**^	−.314^**^	−.521^**^	−.333^**^	−.531^**^	−.430^**^	−.559^**^	−.571^**^	−.519^**^	−.244^*^	−.359^**^	−.355^**^	−.442^**^	−.365^**^	−.439^**^	−.452^**^	−.546^**^
Weight (n = 92)	−.373^**^	−.470^**^	−.376^**^	−.196	−.419^**^	−.314^**^	−.455^**^	−.405^**^	−.495^**^	−.449^**^	−.393^**^	−.197	−.357^**^	−.337^**^	−.294^**^	−.283^**^	−.380^**^	−.415^**^	−.479^**^
BMI (n = 92)	−.225	−.241	−.255^*^	−.063	−.210	−.166	−.257^*^	−.279^*^	−.261^*^	−.179	−.156	−.082	−.218	−.173	−.095	−.132	−.212	−.286^*^	−.257^*^
NSAA (n = 92)	.527^**^	.689^**^	.529^**^	.419^**^	.705^**^	.431^**^	.631^**^	.607^**^	.709^**^	.707^**^	.676^**^	.232^*^	.330^*^	.484^**^	.615^**^	.565^**^	.484^**^	.597^**^	.721^**^
10-m run/walk (*n* = 77)	−.506^**^	−.566^**^	−.182	−.050	−.524^**^	−.057	−.360^**^	−.343^**^	−.593^**^	−.526^**^	−.469^**^	. 074	.067	−.110	−.366^**^	−.605^**^	−.125	−.268^*^	−.578^**^
Gowers (n = 68)	−.586^**^	−.545^**^	−.147	.087	−.475^**^	.052	−.366^**^	−.257	−.601^**^	−.480^**^	−.388^**^	. 066	.086	.042	−.215	−.705^**^	.034	−.172	−.509^**^
4-stair climb (*n* = 68)	−.496^**^	−.592^**^	−.188	.056	−.415^**^	−.026	−.235	−.323^*^	−.599^**^	−.467^**^	−.308^*^	.146	.117	−.145	−.229	−.668^**^	−.047	−.192	−.540^**^
4-stair descend (n = 68)	−.480^**^	−.448^**^	−.241	−.004	−.333^**^	−.020	−.203	−.240	−.527^**^	−.457^**^	−.337^**^	.220	.100	−.120	−.204	−.472^**^	−.032	−.088	−.382^**^
CK (n = 92)	.354^**^	.373^**^	.292^**^	.167	.422^**^	.208	.418^**^	.491^**^	.439^**^	.479^**^	.432^**^	.054	.160	.191	.363^**^	.308^**^	.320^**^	.396^**^	.467^**^

All statistics were evaluated based on Spearman correlation corrected by the Benjamini-Hochberg procedures. * *P* < 0.05, ** *P* < 0.01. *GMa* Gluteus maximus, *GMe* Gluteus medius, *GMi* Gluteus minimus, *IP* Iliopsoas, *TF* Tensor fascia, *OI* Obturator internus, *Pe* Pectineus, *RF* Rectus femoris, *VL* Vastus lateralis, *VI* Vastus intermedius, *VM* Vastus medialis, *Gr* Gracilis, *Sa* Sartorius, *AL* Adductor longus, *AB* Adductor brevis, *AM* Adductor magnus, *St* Semitendinosus, *Sm* Semimembranosus, *BFLH* Biceps femoris long head, *BMI* Body mass index, *NSAA* Northstar ambulatory assessment, *CK* Creatine kinase

The correlation between Mercuri scale of fat infiltration and clinical assessments were shown in Supplementary Table 1. Positive correlations (*P* <0.05) were found between grade of fat infiltration and the participants’ age, height, weight, BMI, 10-m run/walk, Gowers manoeuvre, 4-stair climb, 4-stair descend. Negative correlations (*P* <0.05) were discovered between grade of fat infiltration and NSAA, serum CK level. Most of the correlations between Mercuri scale of fat infiltration and clinical assessments were moderate to strong [r (absolute value) > 0.4, *P* < 0.01].

Supplementary Table 2 presented the results of Mann-Whitney test for individual muscle T1-value and Mercuri scale in DMD patients who used wheelchairs or not. In DMD patients who used wheelchairs or not, there was a significant difference (*P* < 0.001) in T1-value of 19 muscles and Mercuri scale of 18 muscles (except gracilis). It revealed that wheelchair-used patients had lower T1-values and higher score of Mercuri scale in individual muscle than no-wheelchair-used patients.

### Multiple linear regression analysis for the independent association of muscle T1-value and motor function

**Fig. 5:**
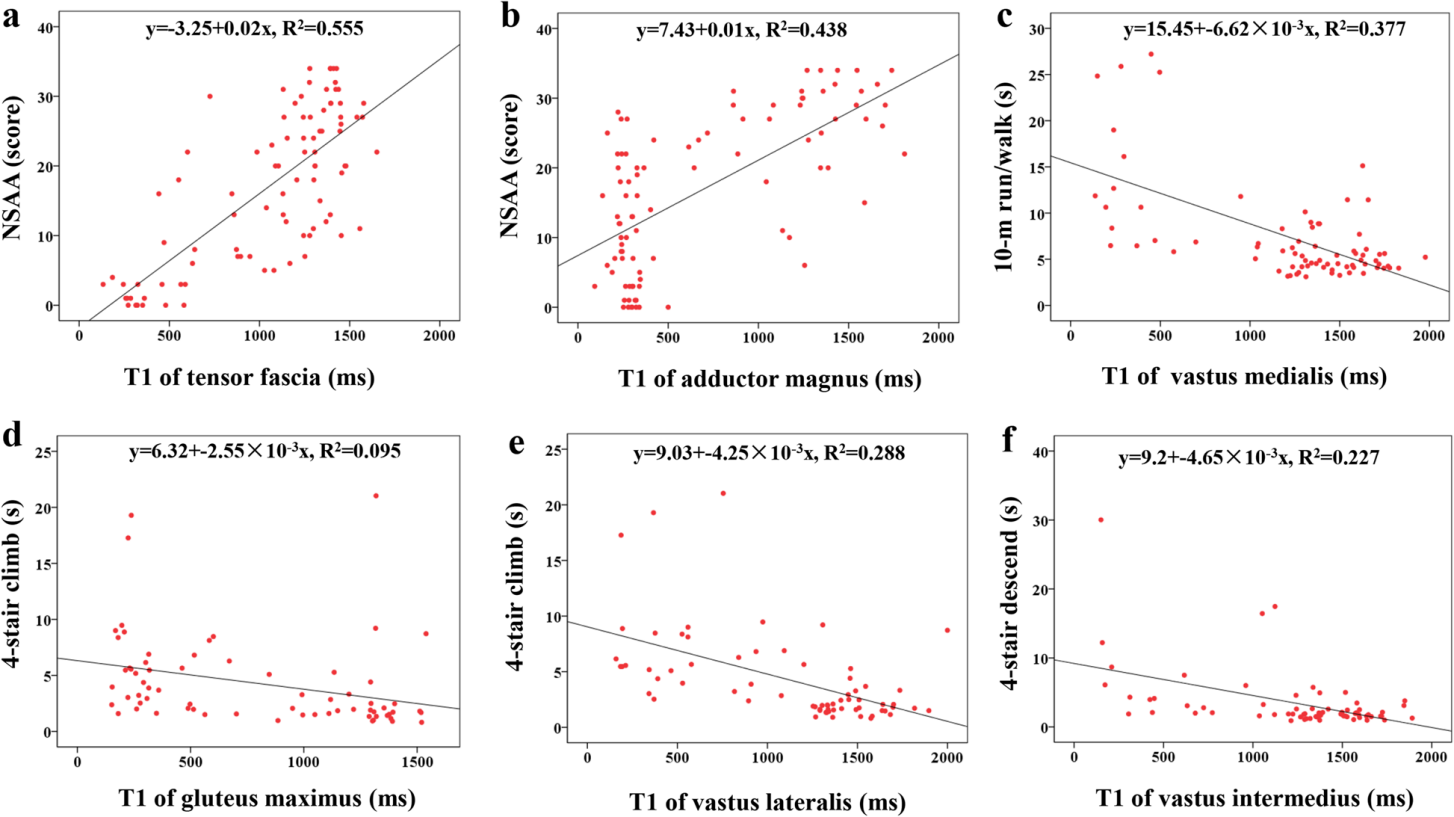
Correlations and regression lines between individual muscle T1-value and clinical motor function tests were presented by scatter plot. The T1-value of tensor fascia and adductor magnus all possessed an independent linear association with NSAA (**a, b**). The T1-value of vastus medialis showed an independent linear association with 10-m run/walk (**c**). The T1-value of gluteus maximus and vastus lateralis all achieved an independent linear relevance with 4-stair climb (**d, e**). The T1-value of vastus intermedius manifested an independent linear relevance with 4-stair descend (**f**)

After excluding no statistically significant independent variables in correlation analysis of muscle T1-value and motor function, we performed the multiple linear regression analysis. In this model, motor function tests were the dependent variables and individual muscle T1-values were the independent variables. The results of multiple linear regression between individual muscle T1-value and clinical motor function tests were shown in Table 4. The T1-value of tensor fascia and adductor magnus possessed an independent association with the NSAA score. The T1-value of vastus medialis showed an independent association with 10-m run/walk. The T1-value of gluteus maximus and vastus lateralis achieved an independent relevance with 4-stair climb. T1-value of vastus intermedius manifested an independent relevance with 4-stair descend. Correlations and regression lines for the above factors were presented by scatter plot (Fig. 5).

**Table 4:** Results of multiple linear regression between individual muscle T1-value and clinical motor function tests

T1-value of muscle	NSAA (*n* = 92)		10-m run/walk (*n* = 77)		Gowers (*n* = 68)		4-stair climb (*n* = 68)		4-stair descend (*n* = 68)	
	***β***	*P*	***β***	*P*	***β***	*P*	***β***	*P*	***β***	*P*
Gluteus maximus	−.107	.348	−.032	.841	−.086	.616	−.343	.048^*^	−.051	.682
Gluteus medius	.033	.815	.248	.152	.069	.685	−.222	.243	−.109	.497
Gluteus minimus	.157	.248	–	–	–	–	–	–	–	–
Iliopsoas	.090	.465	–	–	–	–	–	–	–	–
Tensor fascia	.331	.029^*^	−.276	.096	−.167	.220	−.174	.201	−.175	.166
Obturator internus	−.126	.370	–	–	–	–	–	–	–	–
Pectineus	−.257	.181	−.032	.880	−.228	.158	–	–	–	–
Rectus femoris	−.183	.277	.185	.384			.232	.229		
Vastus lateralis	.131	.497	−.142	.567	−.337	.167	−.618	.018^*^	−.228	.212
Vastus intermedius	.202	.379	−.323	.245	.044	.867	.156	.574	−.476	.001^**^
Vastus medialis	.169	.482	−.142	.033^*^	−.395	.111	−.137	.591	−.237	.317
Gracilis	−.015	.857	–	–	–	–	–	–	–	–
Sartorius	−.064	.599	–	–	–	–	–	–	–	–
Adductor longus	.188	.283	–	–	–	–	–	–	–	–
Adductor brevis	.195	.298	−.209	.196	–	–	–	–	–	–
Adductor magnus	.324	.013^*^	−.051	.778	−.182	.349	−.382	.061	−.099	.447
Semitendinosus	−.088	.587	–	–	–	–	–	–	–	–
Semimembranosus	.109	.539	.144	.408	–	–	–	–	–	–
Biceps femoris long head	−.094	.593	−.119	.582	.115	.591	.245	.270	−.112	.473

Motor function tests were the dependent variables and individual muscle T1-values were the independent variables in this mode. Statistically significant, * *P*-value < 0.05, ** *P*-value < 0.01. “—”, indicating that there were no statistically significant independent variables in Spearman’s correlation analysis of muscle T1-values and motor function, which were not included in the multiple linear regression analysis. NSAA, northstar ambulatory assessment

### Inter-rater reliability of the T1-value ROI measurement

**Fig. 6:**
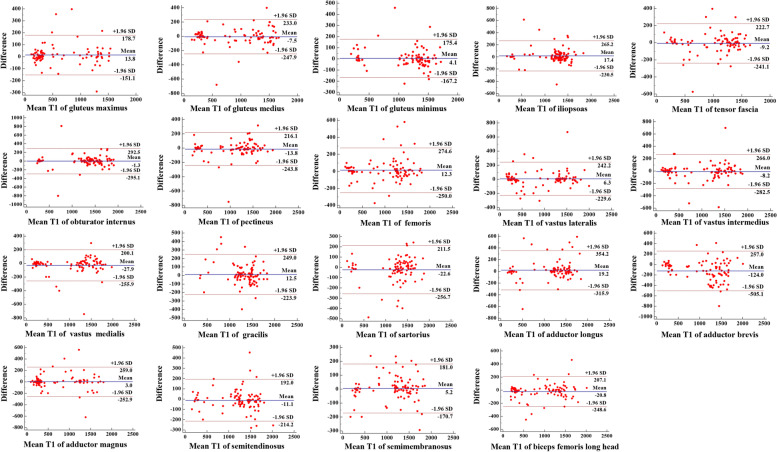
Bland-Altman plots for T1-value region of interest (ROI) measurements in individual muscle of the pelvic girdle and thigh from two independent observers

The Bland-Altman plots and ICC analysis showed excellent inter-rater reliability of T1-value ROI measurement. Bland-Altman plots presented good agreements between the two independent observers (Fig. 6). The range of ICC-intra and ICC-inter was 0.931-0.994 and 0.908-0.985, respectively.

## Discussion

DMD is an X-linked recessive inherited NMD due to the dystrophin gene mutations can lead to partial to complete dystrophin deficiency [23]. Dystrophin-deficient muscles possess a weak structure of the sarcolemma and are vulnerable to contractile injury [3]. Muscle damage is followed by muscle repairment and inflammation, which occur in the early stage of DMD and finally progress to necrosis of muscle fibers and fatty replacement of myofibers [19].

Previous studies demonstrated a characteristic distribution of fat infiltration in pelvic, and thigh muscles in DMD was as follows: in thigh muscles, the adductor magnus were most severely involved, while the gracilis, sartorius, and semitendinosus muscles were relatively spared; the gluteus maximus and gluteus medius were the two most severely affected muscles in pelvic girdle muscles [19, 20, 24, 25]. Our study revealed the same distribution of fat infiltration patterns on T1WI. Consistently, similar muscle-involving and sparing patterns on T1-value results were discovered. The gracilis had the highest T1-value in thigh muscles, followed by the sartorius and semitendinosus; the adductor magnus had the lowest T1-value. The T1-value of the gluteus maximus and gluteus medius was the first and second-lowest among pelvic girdle muscles, separately.

In this study, the T1-value of individual muscle decreased as the grade of fat infiltration increased scored by T1WI. Similar findings were reported by Marty B et al., who discovered lower limb muscle T1-values in BMD and inclusion body myositis (IBM) patients were statistically lower than that of healthy volunteers, and muscle T1-value negatively correlated with intramuscular fat fraction (FF) [16, 17]. Besides, Liu CY et al. found that T1-values of thigh and calf muscles in GNE myopathy decreased with the gradual aggravation of fat infiltration [18]. Interestingly, the muscle T1-values in their study strongly decreased to 318 ± 39.9 ms when muscle tissues were largely or entirely replaced by fat, which was just within the range of our mean T1-values (234.9 to 390.4 ms) of the highest grade of fat infiltration. Taken together, we think T1-mapping can establish a quantitative method for assisting clinical diagnosis, disease severity assessment, and disease progression evaluation in DMD patients, which may reduce or avoid repeated invasive muscle biopsies in clinical trials.

DMD is characterized by progressive, irreversible intramuscular fat infiltration, so FF becomes the main driver of muscle T1 change. Besides, inflammation and fibrosis can also alter T1. Edema/inflammatory and fibrotic processes increase T1, whereas fatty infiltration decreases T1 [15, 17, 26]. The earliest pathological features in DMD include edema/inflammation followed by intramuscular fatty infiltration [27, 28]. Elevated muscle T1 was observed in young DMD patients than healthy controls [29, 30]. This may be due to the fact that, in the early stage of the disease, the effect of edema/inflammation on T1 is greater than the fatty infiltration that begins to occur, even if the two pathological processes occur in parallel. Moreover, this is also considered to correspond to the inflammatory response occurring in the early phase of muscle degeneration and regeneration processes [17]. In the later stage of DMD, dead muscle cells are ultimately replaced by fibro-fatty tissues, especially fat [27]. Even though fibrosis increases T1, there is a strongly decrease in T1 as severe fat-replacement occurs.

The close correlations between Mercuri scale of fat infiltration and clinical assessments in our study were consistent with previously demonstrated results [8, 15, 19, 20]. As we expected, muscle T1-value was also closely related to a series of clinical assessments. The decreasing of muscle T1-values was correlated with the increase of age, height, weight in our study, which was in accord with the known clinical discovery that DMD was an age-dependent and progressive disease [3, 31]. The muscle T1-value correlated with wheelchair use negatively. Wheelchair use meant the DMD patients had entered the last stage of non-ambulatory [7]. Simultaneously the muscles progressed to the most severe fat infiltration and showed the lowest T1-value. As a biochemical marker, CK is a common screening tool for the identification of muscle pathology [32, 33]. Positive correlations were discovered between muscle T1-value and CK in this study, which conformed to the known clinical findings that CK increased significantly during the early stages of the DMD, and then decreased at the later stage of disease because the muscle generating the serum CK was gradually lost [19, 34].

The US Centers for Disease Control and Prevention (CDC) proposed a guideline that the motor function should be monitored routinely every 6 months in DMD patients to judge the disease progression, therapeutic response and adjust the suitable treatment methods [5, 7]. Correlations between motor function and several skeletal muscle MRI measures has been well expounded in many DMD cross-sectional studies [10]. Nevertheless, the correlation between muscle T1-value and motor function have rarely been studied in DMD. Our correlation analysis between individual muscle T1-value and motor function presented exciting results. First, a downward trajectory in motor function may exist with the decrease of skeletal muscle T1-value. Second, among the 19 muscles we selected, the T1-value of 10 muscles (gluteus maximus, gluteus medius, tensor fascia, pectineus, rectus femoris, vastus lateralis, vastus intermedius, vastus medialis, adductor magnus, biceps femoris long head) showed a significant correlation with both NSAA and timed functional tests (*P*<0.05). This is not hard to explain since fat infiltration is considered to be an independent factor related to muscle weakness [13, 20], these 10 muscles have relatively severe fat infiltration (top-10 ranking of mean score of Mercuri scale, simultaneously the last-10 ranking of mean T1-value). Third, T1-value of muscles known as “relatively spared muscles” (especially gracilis and sartorius) in the thigh showed a relatively weak correlation with NSAA score (r = 0.232-0.330, *P*<0.05) and non-correlation with timed functional tests (r = 0.066-0.220, *P*>0.05). This is consistent with the results reported by Ropars J et al. (8) in a systematic review about the correlation between muscle FF and motor function.

Based on these findings and given the heterogeneity of muscles, it is reasonable to deduce that the ability of different muscle T1-value in predicting motor function may vary with different involved patterns. In our results of multiple linear regression, the T1-value of gluteus maximus, tensor fascia, vastus lateralis, vastus intermedius, vastus medialis, and adductor magnus possessed an independent association with NSAA or timed functional tests, which suggests that these muscles may be the “critical” muscles in T1-mapping studies to independently predict the motor function of DMD patients. Previous studies using muscle FF or T2-value to predict motor function revealed the gluteus maximus, biceps femoris long head, quadriceps, or single vastus lateralis were considered to play a more important role in predicting the future loss of ambulation for DMD patients [8, 19, 35, 36]. The “critical” muscles found in our study overlap and differ from previous studies. This may be due to the use of different MRI indices and motor function assessment methods. Although more longitudinal studies are also needed to further elaborate the clinical applications of T1-mapping in predicting motor function, it has shown great potential to be a good candidate.

At last, we also want to discuss the pros and cons of several commonly used fat quantification techniques and the necessity of multimodal MRI evaluation patterns in DMD. The 3-point Dixon has been proved to be a reliable method for measuring intramuscular FF and is considered a useful biomarker of disease severity. In contrast, it may be limited when obvious fat infiltration has appeared at the later stage of disease [8, 15]. ^1^H-MRS has been proposed as an effective method to distinguish early pathologic changes and measure intramuscular FF; unfortunately, MRS data are generally obtained from one sampled area in the muscle, the results can not be extended to the whole muscle [8, 14]. T1-mapping can sample from the entire muscle region. Even in the later stage of disease, the severe intramuscular fat infiltration can also be monitored without restriction. However, the change of tissue water content can cause T1-value variations; if these events, such as inflammation/edema, happen parallel to fatty degenerations, this may represent a bias for FF quantification based on global T1-value measurements [16, 17]. In the next step, a multi-component analysis of the T1-value recovery should be performed to definitely separate water and fat proton signals that can allow the use of water T1-value as an independent biomarker. Given all of that, multimodal MRI is required to comprehensively assess the severity of overall fat infiltration in DMD patients. Moreover, since the variables of imaging indices and fat infiltration may not follow the same disease progression, the most appropriate MRI technique may also vary with different disease stages.

In addition, T2-mapping has been widely utilized in DMD to track disease progression and muscle T2 has shown a significant correlation with motor function [28]. Compared with T2-mapping, T1-mapping is relatively less used in DMD, but it has also shown encouraging results. First, T1-mapping shows great potential for monitoring chronic fatty degenerations of skeletal muscle within the course of NMDs [16–18, 37]. Second, T1 is sensitive not only to the fat infiltration but also other pathological events such as edema/inflammation. Edema/inflammatory process increases T1-value while fat infiltration decreases T1-value. In contrast, both the process of edema/inflammation and fat infiltration increase T2-value. Thus, T1 is helpful to the differentiation of edema/inflammation and fatty infiltration in DMD. Third, T1 is highly correlated with T2 [17, 30], we conjecture that combining T1 and T2 may allow better assessment of pathological events, disease status and disease progression, etc. in the future study of DMD. So it will be interesting to study the application of T1-mapping in DMD.

There were several limitations in our study. First, taking the semi-quantitative Mercuri scale as a reference, we quantitatively evaluated intramuscular fat infiltration by measuring the T1-value. Mercuri scale is subjective and may lead to overestimation or underestimation. The MRI technique such as MRS or Dixon that is used to quantify FF will be included in our next study. Second, although the degree of skeletal muscle edema in DMD patients is relatively mild [9, 20], we have not exclude the influence of inflammation/edema if these events happen parallel to fatty degenerations, which may represent a bias for fat infiltration monitoring based on global T1-value. Third, intramuscular fat infiltration is the main disease process of DMD, yet it also can be observed in other situations like obesity [38, 39]. Other possible non-DMD–related intramuscular fat infiltration factors were not be controlled in our study.

## Conclusion

T1-value generated by T1-mapping of pelvic and thigh muscles decreases as the grade of fat infiltration increased scored by T1WI. Skeletal muscle T1-value can be used as a quantitative biomarker for disease involvement, further to assess the disease severity and predict motor function in DMD.

## Supplementary Information

**Additional file 1.**

## Data Availability

The datasets generated and/or analysed during the current study are not publicly available (because it involves human genetic resources information in our country) but are available from the corresponding author on reasonable request.

## Abbreviations

DMD: Duchenne muscular dystrophy
NMDs: Neuromuscular disorders
CK: Creatine kinase
MRI: Magnetic resonance imaging
BMD: Becker muscular dystrophy
GNE: UDP-N-acetylglucosamine 2-epimerase/N-acetylmannosamine kinase
T1WI: T1 weighted images
MOLLI: Modified look-locker inversion
FOV: Field of view
FA: Flip angle
ROI: Region of interest
BMI: Body mass index
NSAA: NorthStar ambulatory assessment
ICC: Interclass correlation coefficient
IBM: Inclusion body myositis
FF: Fat fraction
MRS: Magnetic resonance spectroscopy
