# Supplementary material for: Utilization of T1-Mapping for the pelvic and thigh muscles in Duchenne Muscular Dystrophy: a quantitative biomarker for disease involvement and correlation with clinical assessments
Source: BMC Musculoskelet Disord. 2022 Jul 16;23:681. doi: 10.1186/s12891-022-05640-y (PMC9288085; doi:10.1186/s12891-022-05640-y)
Supplement: Supplementary file 1 — Additional file 1. [file 12891_2022_5640_MOESM1_ESM.docx]

**Supplementary Table 1**. Spearman correlation between Mercuri scale of individual muscle and clinical assessments

|  | Mercuri scale of individual muscle | | | | | | | | | | | | | | | | | | |
| --- | --- | --- | --- | --- | --- | --- | --- | --- | --- | --- | --- | --- | --- | --- | --- | --- | --- | --- | --- |
|  | GMa | GMe | GMi | IP | TF | OI | Pe | RF | VL | VI | VM | Gr | Sa | AL | AB | AM | St | Sm | BFLH |
| Age (n=92) | .503**^＊＊^** | .688**^＊＊^** | .625**^＊＊^** | .511**^＊＊^** | .590**^＊＊^** | .578**^＊＊^** | .682**^＊＊^** | .605**^＊＊^** | .614**^＊＊^** | .719**^＊＊^** | .656**^＊＊^** | .379**^＊＊^** | .552**^＊＊^** | .519**^＊＊^** | .582**^＊＊^** | .574**^＊＊^** | .563**^＊＊^** | .661**^＊＊^** | .673**^＊＊^** |
| Height (n=92) | .548**^＊＊^** | .563**^＊＊^** | .517**^＊＊^** | .455**^＊＊^** | .558**^＊＊^** | .433**^＊＊^** | .564**^＊＊^** | .533**^＊＊^** | .564**^＊＊^** | .615**^＊＊^** | .565**^＊＊^** | .334**^＊＊^** | .494**^＊＊^** | .426**^＊＊^** | .521**^＊＊^** | .485**^＊＊^** | .446**^＊＊^** | .555**^＊＊^** | .584**^＊＊^** |
| Weight (n=92) | .555**^＊＊^** | .516**^＊＊^** | .492**^＊＊^** | .386**^＊＊^** | .459**^＊＊^** | .421**^＊＊^** | .537**^＊＊^** | .492**^＊＊^** | .463**^＊＊^** | .528**^＊＊^** | .492**^＊＊^** | .371**^＊＊^** | .539**^＊＊^** | .434**^＊＊^** | .425**^＊＊^** | .444**^＊＊^** | .448**^＊＊^** | .519**^＊＊^** | .502**^＊＊^** |
| BMI (n=92) | .343**^＊＊^** | .287**^＊^** | .309**^＊＊^** | .218**^＊^** | .228**^＊^** | .259**^＊^** | .352**^＊＊^** | .334**^＊＊^** | .221**^＊^** | .270**^＊^** | .296**^＊＊^** | .260**^＊^** | .415**^＊＊^** | .294**^＊＊^** | .238**^＊^** | .270**^＊^** | .339**^＊＊^** | .350**^＊＊^** | .264**^＊^** |
| NSAA (n=92) | -.570**^＊＊^** | -.723**^＊＊^** | -.686**^＊＊^** | -.639**^＊＊^** | -.741**^＊＊^** | -.664**^＊＊^** | -.726**^＊＊^** | -.733**^＊＊^** | -.789**^＊＊^** | -.781**^＊＊^** | -.764**^＊＊^** | -.474**^＊＊^** | -.635**^＊＊^** | -.682**^＊＊^** | -.771**^＊＊^** | -.703**^＊＊^** | -.623**^＊＊^** | -.697**^＊＊^** | -.761**^＊＊^** |
| 10-m run/walk (n=77) | .492**^＊＊^** | .600**^＊＊^** | .455**^＊＊^** | .379**^＊＊^** | .611**^＊＊^** | .422**^＊＊^** | .436**^＊＊^** | .582**^＊＊^** | .723**^＊＊^** | .705**^＊＊^** | .641**^＊＊^** | .250**^＊^** | .389**^＊＊^** | .404**^＊＊^** | .650**^＊＊^** | .668**^＊＊^** | .389**^＊＊^** | .441**^＊＊^** | .659**^＊＊^** |
| Gowers (n=68) | .567**^＊＊^** | 504**^＊＊^** | .328**^＊＊^** | .165 | .502**^＊＊^** | .166 | .540**^＊＊^** | .459**^＊＊^** | .643**^＊＊^** | .557**^＊＊^** | .512**^＊＊^** | .175 | .334**^＊＊^** | .198 | .500**^＊＊^** | .681**^＊＊^** | .236 | .304**^＊^** | .509**^＊＊^** |
| 4-stair climb (n=68) | .464**^＊＊^** | .629**^＊＊^** | .344**^＊＊^** | .196 | .478**^＊＊^** | .258**^＊^** | .506**^＊＊^** | .534**^＊＊^** | .732**^＊＊^** | .609**^＊＊^** | .546**^＊＊^** | .171 | .237 | .359**^＊＊^** | .567**^＊＊^** | .705**^＊＊^** | .263**^＊^** | .339**^＊＊^** | .598**^＊＊^** |
| 4-stair descend (n=68) | .508**^＊＊^** | .500**^＊＊^** | .238 | .284**^＊^** | .421**^＊＊^** | .236 | .436**^＊＊^** | .470**^＊＊^** | .630**^＊＊^** | .553**^＊＊^** | .498**^＊＊^** | .182 | .184 | .273**^＊^** | .425**^＊＊^** | .508**^＊＊^** | .188 | .347**^＊＊^** | .487**^＊＊^** |
| CK (n=92) | -.389**^＊＊^** | -.428**^＊＊^** | -.473**^＊＊^** | -.514**^＊＊^** | -.520**^＊＊^** | -.508**^＊＊^** | -.506**^＊＊^** | -.540**^＊＊^** | -.488**^＊＊^** | -.515**^＊＊^** | -.538**^＊＊^** | -.396**^＊＊^** | -.513**^＊＊^** | -.511**^＊＊^** | -.524**^＊＊^** | -.353**^＊＊^** | -.463**^＊＊^** | -.477**^＊＊^** | -.504**^＊＊^** |

All statistics were evaluated based on Spearman correlation corrected by the Benjamini-Hochberg procedures. ＊ *P* < 0.05，＊＊ *P* < 0.01. Gluteus maximus (GMa), Gluteus medius (GMe), Gluteus minimus (GMi), Iliopsoas (IP), Tensor fascia (TF), Obturator internus (OI), Pectineus (Pe), Rectus femoris (RF), Vastus lateralis (VL), Vastus intermedius (VI), Vastus medialis (VM), Gracilis (Gr), Sartorius (Sa), Adductor longus (AL), Adductor brevis (AB), Adductor magnus (AM), Semitendinosus (St), Semimembranosus (Sm), Biceps femoris long head (BFLH). BMI, body mass index; NSAA, northstar ambulatory assessment; CK, creatine kinase.

**Supplementary Table 2.** Mann-Whitney test for individual muscle T1-value and Mercuri scale in DMD patients who used wheelchairs or not

|  |  | Wheelchair (use or not) |
| --- | --- | --- |
| Muscle | Parameters | *P*-value |
| Gluteus maximus | T1-value (ms) | ＜0.001 |
|  | Mercuri scale (1-4) | ＜0.001 |
| Gluteus medius | T1-value (ms) | ＜0.001 |
|  | Mercuri scale (1-4) | ＜0.001 |
| Gluteus minimus | T1-value (ms) | ＜0.001 |
|  | Mercuri scale (1-4) | ＜0.001 |
| Iliopsoas | T1-value (ms) | ＜0.001 |
|  | Mercuri scale (1-4) | ＜0.001 |
| Tensor fascia | T1-value (ms) | ＜0.001 |
|  | Mercuri scale (1-4) | ＜0.001 |
| Obturator internus | T1-value (ms) | ＜0.001 |
|  | Mercuri scale (1-4) | ＜0.001 |
| Pectineus | T1-value (ms) | ＜0.001 |
|  | Mercuri scale (1-4) | ＜0.001 |
| Rectus femoris | T1-value (ms) | ＜0.001 |
|  | Mercuri scale (1-4) | ＜0.001 |
| Vastus lateralis | T1-value (ms) | ＜0.001 |
|  | Mercuri scale (1-4) | ＜0.001 |
| Vastus intermedius | T1-value (ms) | ＜0.001 |
|  | Mercuri scale (1-4) | ＜0.001 |
| Vastus medialis | T1-value (ms) | ＜0.001 |
|  | Mercuri scale (1-4) | ＜0.001 |
| Gracilis | T1-value (ms) | ＜0.001 |
|  | Mercuri scale (1-4) | 0.329 |
| Sartorius | T1-value (ms) | ＜0.001 |
|  | Mercuri scale (1-4) | ＜0.001 |
| Adductor longus | T1-value (ms) | ＜0.001 |
|  | Mercuri scale (1-4) | ＜0.001 |
| Adductor brevis | T1-value (ms) | ＜0.001 |
|  | Mercuri scale (1-4) | ＜0.001 |
| Adductor magnus | T1-value (ms) | ＜0.001 |
|  | Mercuri scale (1-4) | ＜0.001 |
| Semitendinosus | T1-value (ms) | ＜0.001 |
|  | Mercuri scale (1-4) | ＜0.001 |
| Semimembranosus | T1-value (ms) | ＜0.001 |
|  | Mercuri scale (1-4) | ＜0.001 |
| Biceps femoris  long head | T1-value (ms) | ＜0.001 |
|  | Mercuri scale (1-4) | ＜0.001 |
